# Neuroinflammatory signals enhance the immunomodulatory and neuroprotective properties of multipotent adult progenitor cells

**DOI:** 10.1186/s13287-015-0169-z

**Published:** 2015-09-16

**Authors:** Stylianos Ravanidis, Jeroen F. J. Bogie, Raf Donders, David Craeye, Robert W. Mays, Robert Deans, Kristel Gijbels, Annelies Bronckaers, Piet Stinissen, Jef Pinxteren, Niels Hellings

**Affiliations:** Hasselt University, Biomedical Research Institute/Transnational University Limburg, School of Life Sciences, Campus Diepenbeek, Agoralaan building C, 3590 Diepenbeek, Belgium; ReGenesys BVBA, Leuven, Belgium; Department of Regenerative Medicine, Athersys Inc., Cleveland, OH USA

## Abstract

**Introduction:**

Stem cell-based therapies are currently widely explored as a tool to treat neuroimmune diseases. Multipotent adult progenitor cells (MAPC) have been suggested to have strong immunomodulatory and neuroprotective properties in several experimental models. In this study, we investigate whether MAPC are of therapeutic interest for neuroinflammatory disorders such as multiple sclerosis by evaluating their capacities to modulate crucial pathological features and gain insights into the molecular pathways involved.

**Methods:**

Rat MAPC were treated with combinations of pro-inflammatory cytokines that are closely associated with neuroinflammatory conditions, a process called licensing. mRNA expression of immunomodulatory molecules, chemokines and chemokine receptors was investigated. The migratory potential of licensed rat MAPC towards a broad spectrum of chemokines was tested in a Transwell assay. Furthermore, the effect of licensing on the ability of rat MAPC to attract and suppress the proliferation of encephalitogenic T cells was assessed. Finally, neuroprotective properties of rat MAPC were determined in the context of protection from oxidative stress of oligodendrocytes. Therefore, rat MAPC were incubated with conditioned medium of OLN93 cells subjected to sublethal doses of hydrogen peroxide and the gene expression of neurotrophic factors was assessed.

**Results:**

After licensing, a wide variety of immunomodulatory molecules and chemokines, including inducible nitric oxide synthase and fractalkine, were upregulated by rat MAPC. The migratory properties of rat MAPC towards various chemokines were also altered. In addition, rat MAPC were found to inhibit antigen-specific T-cell proliferation and this suppressive effect was further enhanced after pro-inflammatory treatment. This phenomenon was partially mediated through inducible nitric oxide synthase or cyclooxygenase-2. Activated rat MAPC secreted factors that led to attraction of myelin-specific T cells. Finally, exposure of rat MAPC to an in vitro simulated neurodegenerative environment induced the upregulation of mRNA levels of vascular endothelial growth factor and ciliary neurotrophic factor. Factors secreted by rat MAPC in response to this environment partially protected OLN93 cells from hydrogen peroxide-induced cell death.

**Conclusions:**

Rat MAPC possess immune modulatory and neuroprotective properties which are enhanced in response to neuroinflammatory signals. These findings thereby warrant further research to evaluate MAPC transplantation as a therapeutic approach in diseases with an immunological and neurodegenerative component such as multiple sclerosis.

**Electronic supplementary material:**

The online version of this article (doi:10.1186/s13287-015-0169-z) contains supplementary material, which is available to authorized users.

## Introduction

Stem cell transplantation represents a promising therapeutic approach to treat neuroinflammatory and neurodegenerative disorders. By using experimental murine models of neuroinflammatoy diseases, such as experimental autoimmune encephalomyelitis (EAE), traumatic brain injury (TBI) and spinal cord injury (SCI), several studies demonstrated that stem cells reduce neurological deterioration and protect the central nervous system (CNS) from further damage and even stimulate its repair [1–7]. In these studies both adult-derived bone marrow mesenchymal stem cells (MSCs) and newborn CNS-derived neural stem cells (NSCs) provided neurotrophic support and even replaced damaged oligodendrocytes and neurons [3–5]. Of note, the therapeutic action of transplanted cells did not relate to the route of administration—peripheral- or CNS-directed. In addition to the neuroprotective and regenerative potential, the immunomodulatory properties of NSCs and MSCs have been well established [1, 2, 8]. NSCs and MSCs were found to suppress the reactivity of encephalitogenic T cells in the EAE model, thereby likely ameliorating pathological features and clinical symptoms. Collectively, these findings indicate that stem cells can not only halt neuroinflammation but also stimulate CNS repair upon inflammatory neurodegeneration. These properties make them an interesting tool for the treatment of all pathophysiological facets of multiple sclerosis (MS). However, the use of CNS-derived NSCs for autologous transplantation is not a feasible option. Furthermore, although MSCs have been used in clinical trials for autoimmune diseases, the signs of replicative senescence that are demonstrated remain an obstacle for their use as a large-scale clinical product [9–13].

In 2002, another bone marrow-derived stem cell population of mesenchymal origin was initially described, named multipotent adult progenitor cells (MAPC) [14]. Interestingly, in contrast to MSCs, MAPC do not show signs of replicative senescence and possess broader expansion capacities [9–11, 14]. MAPC, in contrast to MSCs, have an extensive differentiation potential towards cell types of all three germ layers depending on the expression levels of pluripotency genes such as *oct-4* [14–16]. Importantly, recent studies indicate that MAPC have neuroprotective and immunosuppressive properties. Rat MAPC (rMAPC) were found to preserve hippocampal cell loss in an animal model of hypoxia-ischemia [17], while human MAPC (hMAPC) stimulated recovery in an animal model of TBI, likely through splenocyte-triggered modulation of microglia phenotype [18–20]. Yet other studies revealed that murine MAPC (mMAPC) attenuate alloreactive T-cell proliferation [21] while hMAPC suppress natural killer (NK) cell proliferation in an indoleamine 2,3-dioxygenase 1 (Ido-1)-dependent manner [22]. Therefore, while MAPC possess intrinsic immune modulating and neuroprotective properties, they show superior features over MSCs, such as the broader expansion rate without any obvious genetic abnormalities [9, 14]. These features make MAPC a more attractive candidate for potential stem cell transplantation therapies in CNS disorders, such as MS, TBI and SCI.

In this study, we defined the therapeutic potential of MAPC in neuroinflammatory diseases. For this purpose, we determined the basal immunomodulatory, migratory, chemoattractive and neuroprotective features of rMAPC. Next, the impact of pro-inflammatory and neurodegenerative stimuli on the physiology of rMAPC was assessed. It was previously reported that a pro-inflammatory milieu markedly alters the physiology of stem cells (a process called ‘licensing’) [23–26]. rMAPC were licensed with combinations of interferon gamma (IFNγ), tumor necrosis factor alpha (TNFα) and interleukin-1 beta (IL1β). These cytokines are highly expressed in the brain parenchyma and cerebrospinal fluid in CNS neuroinflammatory diseases and play a crucial role in their pathophysiology. Particularly, IFNγ secreted by Th1 cells leads to the activation of other immune cells whereas TNFα and IL1β secreted by activated macrophages and microglia lead to direct destruction of myelin sheath and oligondendrocytes [27–30]. We show that unstimulated rMAPC possess immunomodulatory and neuroprotective properties which are further enhanced when challenged with neuroinflammatory signals. Specifically, rMAPC suppressed autoreactive T-cell proliferation and protected oligodendrocytes from hydrogen peroxide (H_2_O_2_)-induced damage. We further present that licensing increases the capacity of rMAPC to attract T cells, while they themselves adopt an enhanced migratory profile. Collectively these findings show that rMAPC, when challenged *in vitro* with signals that are overexpressed in a neuroinflammatory environment, acquire a phenotype which may limit disease activity *in vivo*.

## Materials and methods

### rMAPC culture and inflammatory licensing

Lewis rMAPC and culture medium were provided by ReGenesys (Leuven, Belgium). rMAPC were isolated and maintained according to a previously described protocol [15]. Briefly, bone marrow from tibiae and femur from Lewis rats was excised and flushed using phosphate-buffered saline (PBS; Lonza, Verviers, Belgium). The single cell suspension was centrifuged and washed using rMAPC medium. rMAPC medium consisted of 60 % Dulbecco’s modified Eagle's medium (DMEM; Gibco, Life Technologies Europe B.V., Gent, Belgium) low glucose (1 g/l) , 40 % MCDB-201 medium (pH 7.2), 1× linoleic acid-bovine serum albumin (BSA), 10^−4^ M L-Ascorbic acid, 0.05 μM dexamethasone, 55 μM 2-mercapto-ethanol (all from Sigma Aldrich, Diegem, Belgium), 100 IU/ml penicillin and 100 μg/ml streptomycin (Invitrogen, Life Technologies Europe B.V.), 1× insulin-transferrin-selenium (Lonza), 10 ng/ml mouse epidermal growth factor, 10 ng/ml recombinant human platelet-derived growth factor (R & D systems, Abingdon, United Kingdom), 2 % fetal bovine serum (Hyclone, EU approved, Cat CH30160.03), and 10^3^ units/ml mouse leukemia inhibitory factor (Millipore). Cells were seeded in fibronectin (10 ng/ml; Sigma Aldrich) coated plates for 1 month in increasing densities. After 1 month of culturing, negative control selection was performed to eliminate CD45^+^ hematopoietic cells. The resulted cell fraction was subcloned until a homogeneous population of small, spindle-shaped cells remained. The cells with MAPC morphology were maintained in culture in order for colonies to be formed. Frozen stock was created with cells in medium containing rMAPC medium, fetal bovine serum and dimethyl sulfoxide (DMSO). Cells were maintained in T175 flasks (Cellstar, Greiner Bio-One, Vilvoorde, Belgium) or petri dishes (Nunc, VWR, Leuven, Belgium) according to the purposes needed and maintained at 37 °C/5 % O_2_. All experiments were performed with rMAPC that reached 10 population doublings maximum.

To assess the impact of pro-inflammatory treatment (licensing) on rMAPC properties, cells were treated with 100 ng/ml of combinations of rat recombinant cytokines (IFNγ + TNFα, IFNγ + IL1β, TNFα + IL1β; all from Peprotech, London, UK) or PBS as vehicle control for 12 or 24 hours. Specific licensing incubation times are described in each section.

### Colony forming unit fibroblast assay

Cells were seeded as 10 cells/cm^2^ in six-well plates. Medium was changed every 2 days. After 12 days, medium was removed and cells were fixed with 4 % paraformaldehyde (PFA) for 20 minutes and then were washed twice with PBS. A solution of 0.5 % crystal violet (Sigma Aldrich) in methanol was added for 30 minutes. Following the incubation time, cells were washed three times with PBS, rinsed with tap water and allowed to air dry before measuring the colonies. To explore the impact of licensing on the ability of rMAPC to form colonies, cells were pre-treated for 24 hours with the three combinations of cytokines and vehicle.

### Flow cytometry

Cells were detached with 0.25 % trypsin (Gibco), harvested and washed with fluorescence-activated cell sorting (FACS) buffer (PBS supplemented with 2 % fetal calf serum (FCS; Gibco)) and incubated in the dark for 30 minutes at 4 °C with surface antibodies. Phenotypic analysis of the cells was performed using CD11b/c, CD31 (BD Biosciences, Erembodegem, Belgium), CD44 (Immunotools, Friesoythe, Germany), CD80, CD86 (eBioscience, Vienna, Austria), RT-1a and RT-1b (Biolegend, San Diego, CA, USA). Cells were positive for CD44 and RT-1a, expressed low to negligible levels of CD80, while they were completely negative for CD11b/c, CD31, CD86 and RT-1b.

### Generation of myelin basic protein-specific T cells

Myelin basic protein (MBP)-specific T cells were isolated as described previously [31]. Briefly, 8-week-old female Lewis rats (Janvier, France) were injected subcutaneously with a 0.1 ml solution of 250 μg/ml guinea pig MBP, 2.5 mg/ml H37RA heat-killed mycobacterium tuberculosis (Difco, Detroit, USA) and 60 μl Complete Freund’s adjuvant (Sigma Aldrich) in both hind paws. Ten days post-immunization, popliteal and inguinal lymph nodes were removed and single cell suspensions were obtained by grinding the tissues through a 70 μm cell strainer with a syringe plunger. The isolated cells were seeded initially in T cell medium consisting of RPMI-1640 medium, 1 % penicillin-streptomycin mixture, 1 % non-essential amino acids, 1 % sodium pyruvate (all from Invitrogen, Life Technologies Europe B.V.), 20 μM 2-mercapto-ethanol (Sigma Aldrich) supplemented with 2 % heat-inactivated autologous serum and 33 μg/ml MBP. After 48 hours, T cells were collected, washed and seeded in T cell medium supplemented with 10 % FCS (Gibco) and 6.5 % CAS medium (supernatant of Concanavalin A (Sigma Aldrich) activated spleen cells) for another 48 hours. Next, cells were collected, washed and seeded in T cell medium supplemented with 10 % FCS for 3 days. All animal experiments were approved by the Ethical Committee for Animal Experiments of Hasselt University.

### Co-cultures

Prior to co-culture with rMAPC, T cells were labeled with 4 μM carboxyfluorescein diacetatesuccinimidyl ester (CFSE; Invitrogen) at a concentration of 2 × 10^7^ cells/ml in PBS/0.1 % BSA (Millipore, Merck Chemicals N.V./S.A., Overijse, Belgium) solution. CFSE-labeled T cells (7.5 × 10^4^ cells/ well) were seeded alongside rMAPC in ratios ranging from 1:0.5 to 1:2 (T cells/rMAPC). Irradiated thymocytes (7.5 × 10^4^ cells/well, 3000 rad) were added to each well as antigen-presenting cells. The medium of the co-cultures consisted of a 1:1 mixture of rMAPC medium and T cell medium with 2 % autologous serum supplemented with 10 μg/ml MBP. To explore the effect of licensing on their suppressive capacity, rMAPC were pre-treated with the three combinations of cytokines and vehicle for 12 hours prior to the co-culture. To define the involvement of nitric oxide (NO), cyclooxygenase (COX)-2 and Ido-1 in suppressive activity, respectively 1.5 mM L-N^G^-monomethyl arginine citrate (L-NMMA;VWR), 10 μM indomethacin (Sigma Aldrich) and 200 μM 1-methyl-L-tryptophan/1-methyl-D-tryptophan (1-Mt-L and 1-Mt-D; Sigma Aldrich) were added at day 0 and day 2 of the co-culture. After 4 days, flow cytometry was used to assess proliferation and cell death of lymphocytes. Therefore, cells were stained with phycoerythrin-conjugated mouse anti-rat CD3 (eBioscience) and 7 aminoactinomycin D (7AAD; BD Biosciences). T-cell proliferation was determined based on CFSE dye dilution of CD3^+^7AAD^−^ cells using flow cytometry (FACSCalibur).

### Migration assays

To explore the chemoattractive properties of rMAPC, we seeded MBP-specific T cells (2.5 × 10^5^ cells/insert) in the upper chamber of a transwell plate with a 5-μm pore size (Sigma Aldrich). In the bottom chamber supernatant of (licensed) rMAPC was placed. To obtain the supernatants, rMAPC were seeded in a mixture of media (30 % MAPC medium, 70 % RPMI-1640) and licensed for 24 hours with respective cytokine combinations. The supernatant was aspirated and filtered through a 0.45-μm filter. Following 4 hours of culture at 37 °C, migrated cells were collected from the bottom chamber and counted using a hemocytometer. Non-conditioned medium served as negative control.

To explore the functionality of chemokine receptors expressed by rMAPC, rat recombinant CCL2, CCL5, CX_3_CL1, CXCL10 and CXCL12α (100, 250 and 500 ng/ml, all from Peprotech) were administered in the bottom chamber of a transwell plate with an 8-μM pore size (Sigma Aldrich). Cells were seeded in the upper chamber at a concentration of 5 × 10^4^ cells/insert and allowed to migrate for 16 hours. Recombinant chemokines were diluted in DMEM-low glucose 1 g/l (Gibco). DMEM (glucose 1 g/l) was used as negative control. As a positive control, rMAPC medium (ReGenesys) was used, as it was optimized to be more effective than the more traditionally used FCS containing solutions (20 % FCS in DMEM 1 g/l solution (see Additional file 1)). To explore the impact of inflammatory conditions on the migration profile of rMAPC, cells were licensed for 12 hours before allowing them to migrate. For the quantification of the migrated fraction we used a protocol by Bronckaers et al. [32] with minor modifications. Briefly, cells on both sides of the insert were fixed with 4 % PFA solution for 20 minutes. Following one washing step with PBS, cells were incubated with 0.1 % crystal violet (Sigma Aldrich) solution in ethanol for 10 minutes at room temperature. Next, cells on the top side of the insert were removed with a cotton swab and an additional washing step with PBS was performed. Wells were allowed to air dry; thereafter, two pictures from each well were taken. Finally, the migrated fraction was analyzed using ImageJ software and expressed as percentage of the total covered area [33].

### OLN93 cell culture, generation of conditioned media and protection assays

The OLN93 cell line was a kind gift from Prof. Dr. C. Richter-Landsberg (University of Oldenburg). Cells were cultured in DMEM high glucose (Sigma Aldrich) supplemented with 10 % FCS and 1 % penicillin-streptomycin mixture at 37 °C/10 % CO_2_. Conditioned media from rMAPC were prepared according to the protocol of Isele et al. [34] with minor modifications. An illustration of the protocol is depicted in Additional file 2. Briefly, OLN93 were seeded in a 96-well flat bottom plate (Greiner Bio-One) (5 × 10^4^ cells/well) and then treated with 1 mM H_2_O_2_ to induce sublethal cell damage. After 2 hours, H_2_O_2_ was removed and OLN93 were allowed to condition fresh medium for 24 hours (OLN-CM_H2O2_). Non-damaged OLN93 cells provided the OLN-CM. The OLN-CM_H2O2_ and OLN-CM were applied to rMAPC in a 96-well flat bottom plate (5 × 10^4^ cells/well) for 18 hours resulting in double-conditioned media (DCM). These media are designated as DCM_H2O2_ and DCM_null_, respectively. rMAPC treated with OLN-CM_H2O2_ and OLN-CM were also processed for RNA extraction (5 × 10^5^ cells/well per 24-well plate) to evaluate alterations in gene expression levels of neurotrophic factors. All the aforementioned conditioned media were collected and filtered through a 0.45-μm filter.

OLN-93 cells were allowed to adhere in a 96-well flat bottom plate (5 × 10^4^ cells/well) and exposed to DCM_H2O2_ and DCM_null_ for 6 hours (see Additional file 2). Subsequently, cells were subjected to H_2_O_2_-induced oxidative stress (1 mM, 1.5 mM and 2 mM H_2_O_2_ for 24 hours). OLN93 cell viability was determined using the MTT assay.

### Gene expression analysis

rMAPC licensed for 12 hours and rMAPC treated with OLN93-derived media (OLN-CM_H2O2_ and OLN-CM) for 18 hours were processed for gene expression analysis. Cells were detached, centrifuged and stored in lysis buffer of RNeasy mini kit (QIAGEN, Venlo, The Netherlands) at −80 °C until later use.

RNA was isolated with the RNeasy mini kit (QIAGEN) and was reversely transcribed into complementary DNA (cDNA) using the Quanta kit (VWR, Leuven, Belgium) following the manufacturer’s instructions. cDNA was subsequently used for semi quantitative real-time polymerase chain reaction (RT-PCR). Quantitative PCR reactions were performed with a StepOnePlus™ Real-Time PCR System (Applied Biosystems) in micro-AMP Fast Optical 96-well reaction plates in a total volume of 10 μl per reaction. The reaction mix contained 1× Fast SYBR green master mix (Applied Biosystems), 10 mM of each primer (designed with Primer 3 [35]; Eurogentec, Liege, Belgium), nuclease-free water and 12.5 ng of cDNA template. The amplification protocol used was the following: 20 seconds at 95 °C, followed by 40 cycles of 3 seconds at 95 °C and 30 seconds at 60 °C, and subsequent melting-curve analysis. All the primer sequences used are listed in Additional file 3. Relative quantification of gene expression was calculated using the 2^-ΔΔCt^ method [36] and data were normalized to the most stable reference genes for each experiment according to geNorm [37]. For visualization of mRNA transcript presence, PCR amplified products (Hoffmann-La Roche, Basel, Switzerland) were separated in a 1.5 % agarose gel and were visualized with ethidium bromide.

### Nitrite formation and cytokine release

Supernatants of licensed rMAPC (24 hours) and co-cultures were collected and release of NO was determined using the Griess reagent system (Promega, Leuven, Belgium) following the manufacturer’s instructions. Absorbance was measured at 550 nm using a microplate reader (Bio-Rad Benchmark, Bio-Rad Laboratories, Hercules, CA, USA).

IFNγ release was measured in the co-culture supernatant using Rat IFNγ enzyme-linked immunosorbent assay (Peprotech) following the manufacturer’s instructions. Absorbance was measured at 415 nm.

### Cell viability assay (MTT)

Cell survival and proliferation was assessed with MTT assay (Sigma Aldrich). Briefly, 12.5 μl MTT (3-(4,5)-dimethylthiazol-(−z-y1)-3,5-diphenytetrazoliumronide) dissolved in 100 μl medium per well was added for 4 hours at 37 °C. After incubation, MTT was removed and a mixture of 25 μl glycine and 150 μl DMSO/well was added and the absorbance was determined at 540 nm.

### Statistical analysis

Data were analyzed with the GraphPad Prism version 5.00 for Windows, (GraphPad Software, San Diego California USA [38]) and are presented as mean ± SEM. D’Agostino and Pearson omnibus normality test was used to test normal distribution. Parametrical data were analyzed using unpaired student *t* test or one way analysis of variance followed by Dunnett multiple comparisons test. Data that did not follow normal distribution were analyzed using Mann Whitney and Kruskal-Wallis followed by Dunns multiple comparison test. Differences with *P* value ≤0.05 were considered significant.

## Results

### *In vitro* licensing does not affect the viability and colony forming ability of rMAPC

**Fig. 1:**
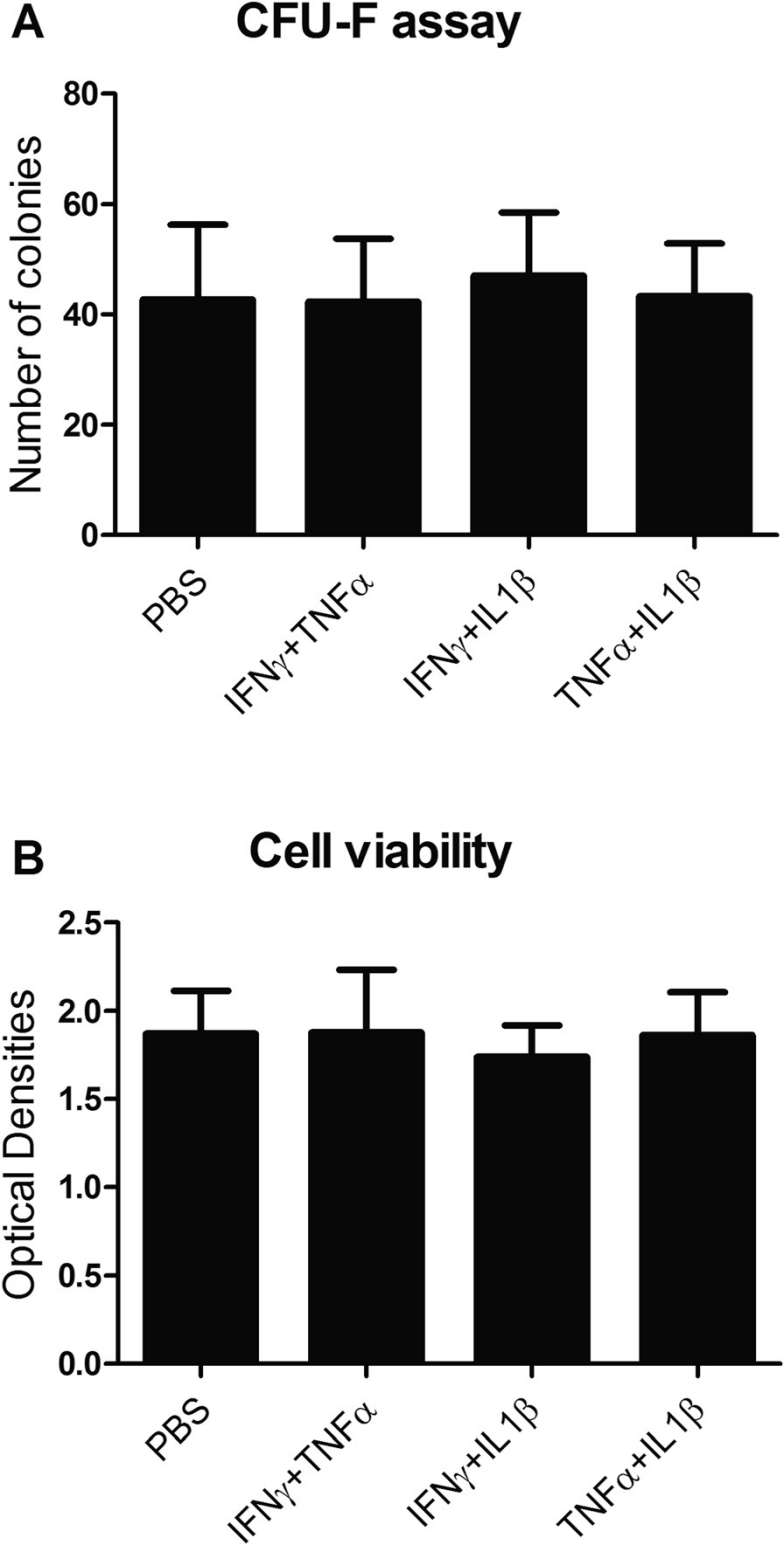
Inflammatory cytokine treatment is not toxic and does not affect colony formation of rMAPC. **a** rMAPC were tested for the formation of colonies following exposure to combinations of inflammatory cytokines (n = 3 experiments, duplicates per experiment). The total number of colonies is shown. **b** Cell viability was measured after 24-hour treatment with combinations of pro-inflammatory cytokines. Optical density values are depicted (n = 4 experiments, triplicates per experiment). *CFU-F* colony-forming unit-fibroblasts, *IFN* interferon, *IL* interleukin, *PBS* phosphate-buffered saline, *TNF* tumor necrosis factor

When stem cells are to be used in treating inflammatory diseases, they will be challenged by a high pro-inflammatory environment. To evaluate whether inflammatory conditions lead to alterations of basic biological functions, we assessed cell viability and colony formation ability following in vitro treatment with combinations of IFNγ, TNFα and IL1β. We show that the viability of rMAPC was not affected after 24 hours treatment (Fig. 1a). Similar, the ability of rMAPC to form colonies was not hampered following pre-treatment with pro-inflammatory cytokines (Fig. 1b). These findings indicate that an inflammatory environment does not affect basic biological functions of rMAPC.

### *In vitro* licensing leads to upregulation of immunomodulatory genes

**Fig. 2:**
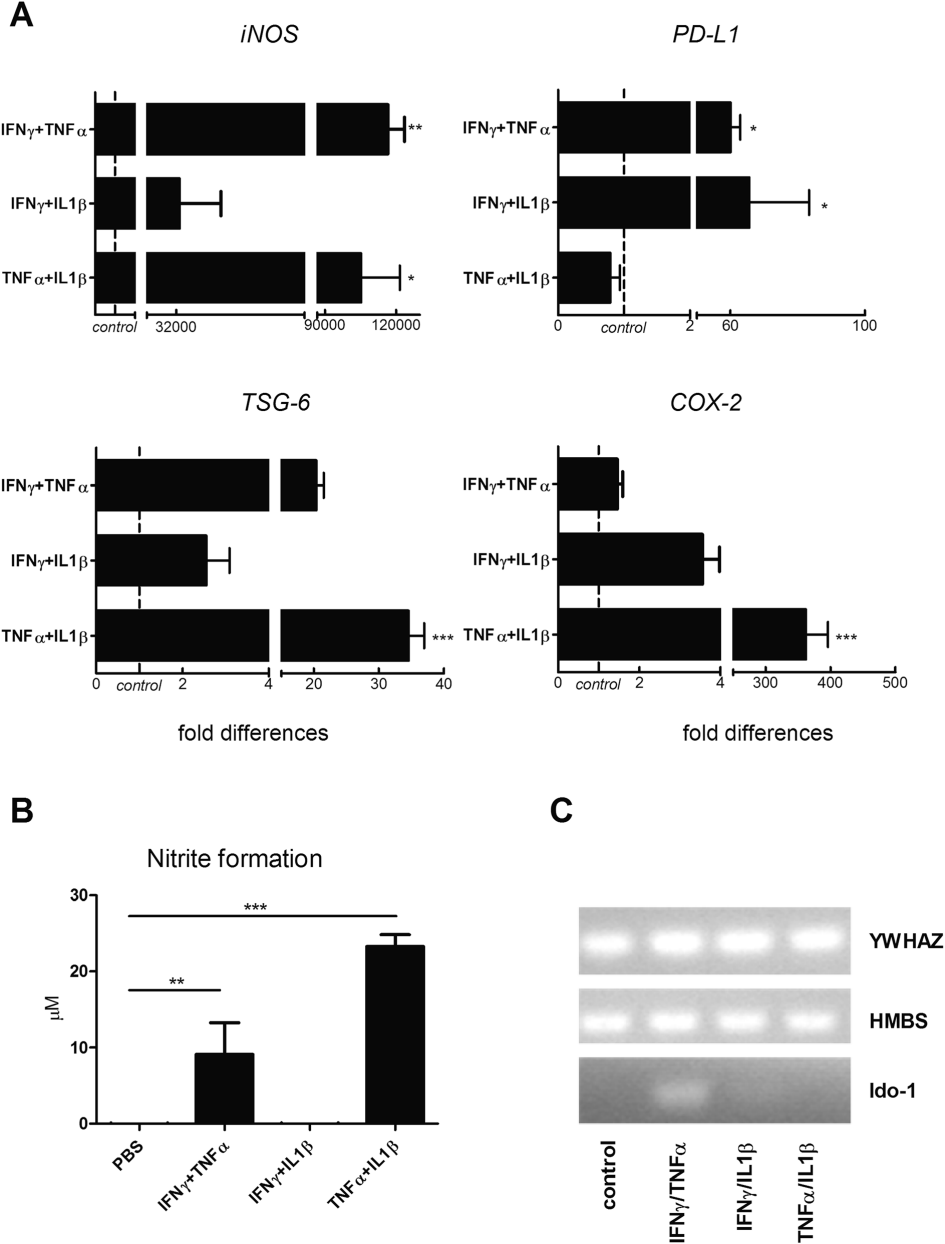
Pro-inflammatory cytokines induce the upregulation of immune modulatory genes by rMAPC. **a** Gene expression of immunomodulatory molecules by rMAPC following treatment with respective inflammatory cytokines. Fold differences compared to control (PBS treated, *dotted line*) are shown. Values represent mean ± SEM from five independent experiments. **b** Measurement of nitrite in the culture supernatant of rMAPC. Concentration of nitrite is shown (μM). Mean values (±SEM) from four independent experiments. **c** Expression of Ido-1 following 12 hours treatment with respective cytokines. YWHAZ and HMBS were used as loading controls. Representative experiment out of n = 5 performed. Non-parametrical Kruskal Wallis multiple group comparison test was used followed by Dunns test for differences between groups. Significant differences with the control condition are indicated with asterisks: *P ≤ 0.05, ** *P* ≤ 0.01, ***P ≤ 0.001. *COX* cyclooxygenase, *HMBS* hydroxymethylbilane synthase, *IFN* interferon, *ido-1* indoleamine 2,3-dioxygenase 1, *IL* interleukin, *iNOS* inducible nitric oxide synthase, *PBS* phosphate-buffered saline, *PD-L1* programmed death-ligand 1, *TNF* tumor necrosis factor, *TSG* TNF stimulated gene, *YWHAZ* 14-3-3 protein zeta/delta

The immune suppressive and neuroprotective features of MSCs are enhanced in the context of neuroinflammatory conditions [23]. We investigated whether the expression of immunomodulatory molecules of rMAPC is changed upon in vitro challenge with selected pro-inflammatory cytokines. Inducible nitric oxide synthase (iNOS), Ido-1, COX2, programmed death-ligand 1 (PD-L1) and TNF-stimulated gene 6 protein (TSG-6) have been proposed as possible mechanisms of immune suppression among others [39–43]. *iNOS* mRNA levels were significantly upregulated in rMAPC following IFNγ + TNFα and TNFα + IL1β treatment (Fig. 2a). In line with this, NO levels increased in the culture medium (Fig. 2b). TNFα + IL1β induced the upregulation of *TSG-6* and *COX-2* mRNA levels. IFNγ was the main inducer of *PD-L1* as TNFα + IL1β did not show any effect (Fig. 2a). *Ido-1* was only detected after IFNγ + TNFα treatment (Fig. 2c). *COX-1* was decreased following TNFα + IL1β treatment while heme oxygenase 1 (*HO-1*) expression was not altered under any of the treatments (see Additional file 4).

Hepatocyte growth factor (HGF) has been suggested to be responsible for neuroprotective and immunomodulatory features of murine MSCs in EAE [44]. In our study, we detected negligible levels of *HGF* in rMAPC in all conditions tested.

The upregulation of inflammatory cytokines by stem cells could potentially counteract their immune suppressive role and enhance inflammation [27–29]. We found a minimal but significant upregulation of *IFNγ* and *TNFα* after IFNγ + IL1β and TNFα + IL1β treatment, respectively. *IL-6* expression was not affected under any of the cytokine combinations (see Additional file 4).

Together, these results suggest that rMAPC, when encountering a pro-inflammatory environment, are triggered to secrete a variety of immunomodulatory molecules.

### rMAPC suppress proliferation of encephalitogenic T cells

**Fig. 3:**
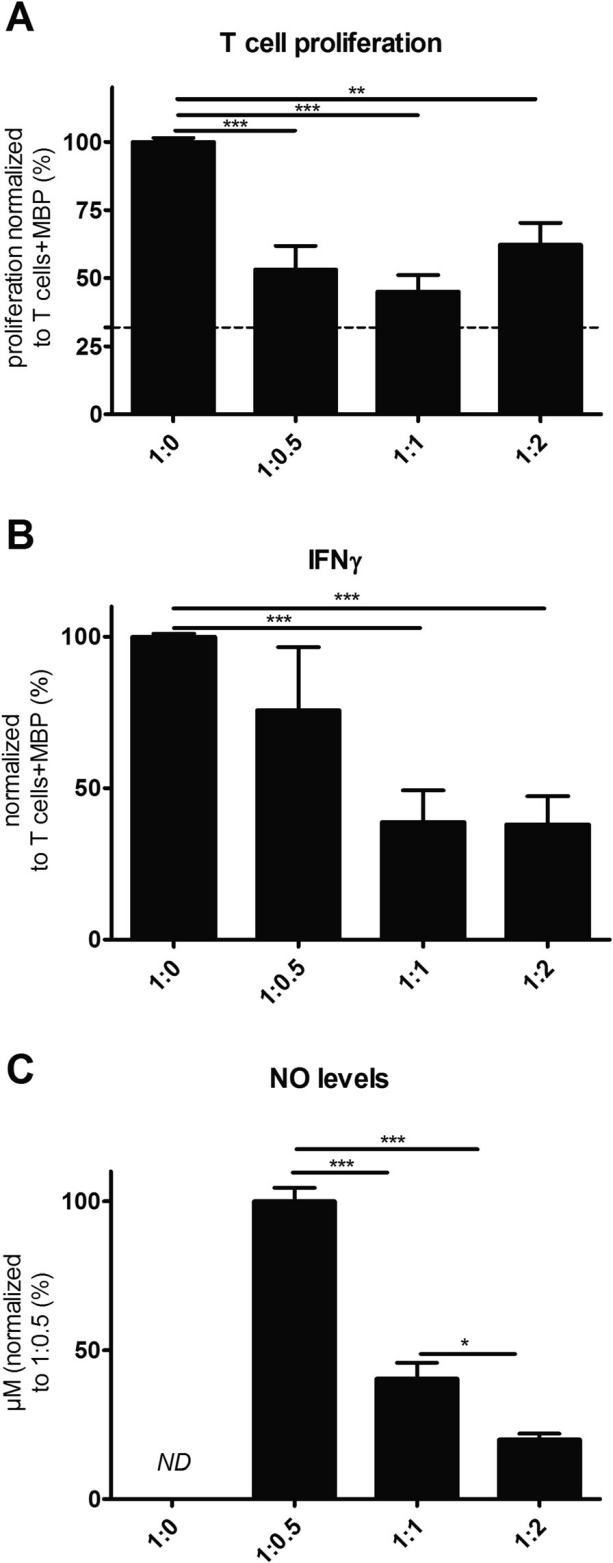
rMAPC suppress antigen-specific T-cell proliferation and IFNγ production. **a** Co-culture of MBP-specific T cells with rMAPC in increasing ratios (1:0.5 to 1:2). Results are shown as percentage of proliferation normalized to the positive control (1:0, T cells + MBP). Mean values ± SEM are from three independent experiments, with triplicates per experiment. Negative control (T cells without MBP) is shown as a *dotted line*. **b** IFNγ levels measured in the co-culture supernatants with enzyme-linked immunosorbent assay. Results are presented as percentages normalized to the positive control (1:0, T cells + MBP). Mean values ± SEM are from three experiments, with triplicates per experiment. **c** Analyses of NO levels in co-culture supernatants through the measurement of nitrite. Results are shown as percentages normalized to the lowest ratio in the co-culture (1:0.5). Mean values ± SEM are from three independent experiments, with triplicates per experiment. Nitrite was not detected (ND) in the positive control (1:0, T cells + MBP), or in thymocytes alone + MBP (not shown). Non-parametrical Kruskal Wallis multiple group comparison test was used followed by Dunns test for differences between groups. Significant differences with the control condition are indicated with asterisks: *P ≤ 0.05, **P ≤ 0.01, ***P ≤ 0.001. *IFN* interferon, *MBP* myelin basic protein, *NO* nitric oxide

To elucidate whether rMAPC are suppressive towards T cells, we performed co-culture experiments with MBP-specific T cells as a model of encephalitogenic T cells. MBP-reactive T cells are evident in diverse neurological disorders such as MS, TBI, SCI and stroke [45–47]. We demonstrate that rMAPC potently suppressed T-cell proliferation in response to MBP even at the lowest ratio (1:0.5). Increasing the number of rMAPC did not further enhance the inhibition of T-cell proliferation (Fig. 3a). The observed effect was not due to cell death, as the percentage of CD3^+^7AAD^+^ cells was not altered (data not shown). In addition, a decrease in IFNγ was detected when increasing amounts of rMAPC were added (Fig. 3b). Of note, NO also decreased in a similar way, in line with the observation that IFNγ induces release of NO by MAPC (Fig. 2a, b). Activated T cells or thymocytes alone did not produce NO (Fig. 3c).

### Inflammatory treatment enhances the suppression of myelin-specific T-cell proliferation

**Fig. 4:**
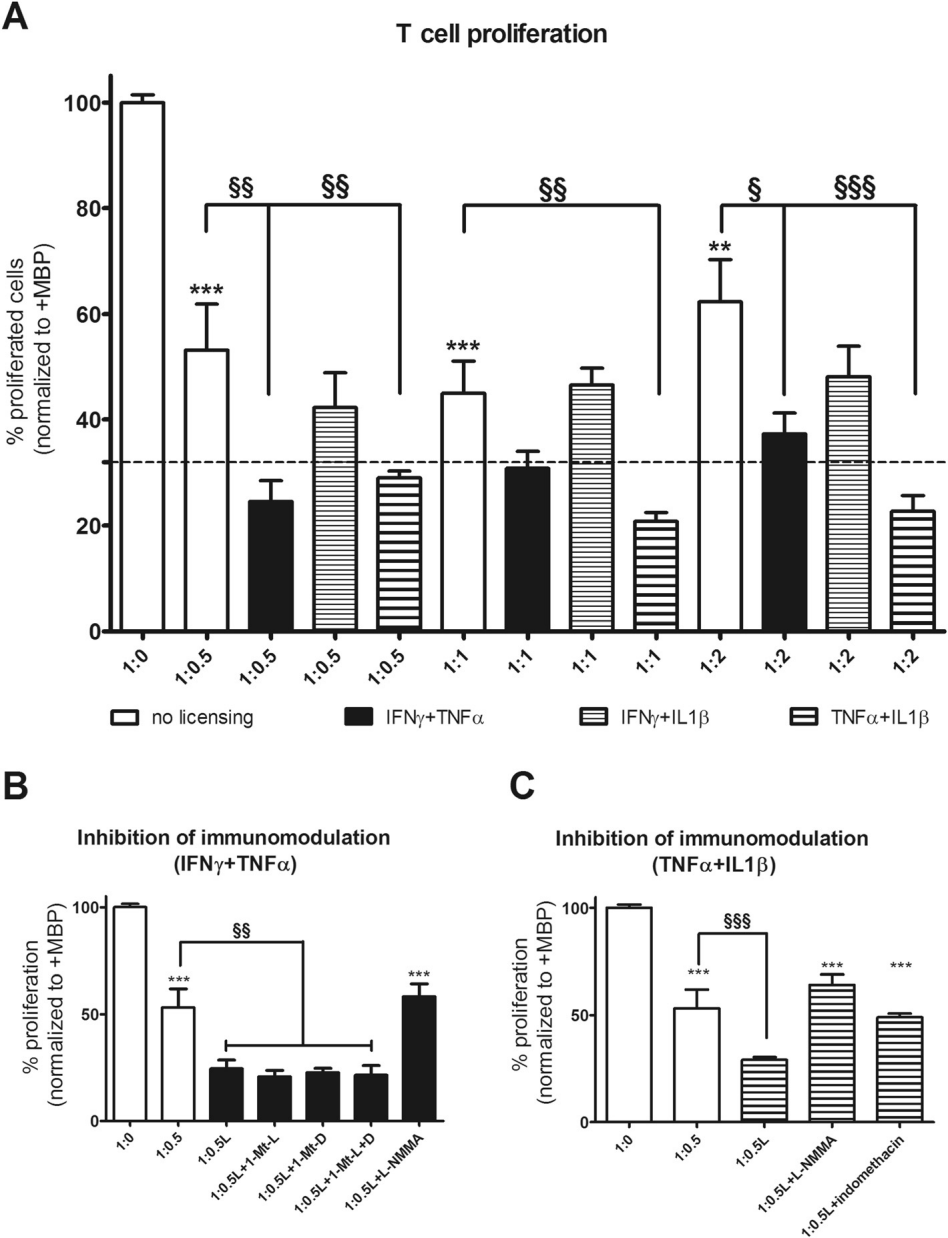
Inflammatory licensing of rMAPC leads to enhanced T-cell suppression. **a** Co-cultures of T cells with rMAPC in the presence of MBP, where rMAPC were either pre-treated or not (*white bars*) with different combinations of pro-inflammatory cytokines (IFNγ + TNFα, IFNγ + IL1β, TNFα + IL1β). Negative control (T cells without MBP) is shown as a *dotted line*. Asterisks indicate statistical significance compared to the positive control (1:0, T cells + MBP). Paragraph signs (§) indicate the statistical significance between the pre-treated (*assigned bars*) and non-treated (*white bars*) corresponding ratios. **b** Application of inhibitors in the co-culture (1:0.5 ratio) after IFNγ + TNFα pre-treatment (*black bars*). Significant differences compared to the positive control (1:0, T cells + MBP) are shown with asterisks. Paragraph signs (§) show differences with the non-pre-treated 1:0.5 ratio (*white bar*). **c** Application of inhibitors in the co-culture (1:0.5 ratio) after TNFα + IL1β pre-treatment (*striped bars*). Significant differences compared to the positive control (1:0, T cells + MBP) are shown with asterisks. Paragraph signs (§) show differences with the non-pre-treated 1:0.5 ratio (*white bar*). Results are shown as mean percentages ± SEM of proliferation normalized to the positive control (1:0, T cells + MBP), from three independent experiments, with triplicates per experiment. Data were analyzed with non-parametrical Kruskal Wallis multiple group comparison test, followed by Dunns for differences between the groups. *P ≤ 0.05, **P ≤ 0.01, ***P ≤ 0.001; ^§^ *P* ≤ 0.05, ^§§^ *P* ≤ 0.01, ^§§§^ *P* ≤ 0.001. *1-Mt-D* 1-methyl-dextro-tryptophan, *1-Mt-L* 1-methyl-levo-tryptophan, *IFN* interferon, *IL* interleukin, *L-NMMA* L-N^G^-monomethyl arginine citrate, *MBP* myelin basic protein, *TNF* tumor necrosis factor

Next, we investigated whether the pro-inflammatory cytokine treatment enhances the suppressive properties of rMAPC towards MBP-specific T cells in line with previous findings for MSCs [40, 43]. Inhibition of antigen-specific T-cell proliferation was enhanced when cells were pre-licensed with either IFNγ + TNFα or TNFα + IL1β. Of note, pre-licensed rMAPC completely inhibited the antigen-specific T-cell proliferation reaching the negative control levels. For IFNγ + IL1β, no increased suppression was found (Fig. 4a).

To identify the mechanisms involved, we applied selective inhibitors for immune modulatory molecules, taking into consideration the upregulation of immunomodulatory genes reported in this study (Fig. 2a). Specifically, we used inhibitors for iNOS (L-NMMA), COX-2 (indomethacin) and Ido-1 (two isoforms; 1-Mt-L, 1-Mt-D). Blockade of NO reversed the observed enhancement of T-cell suppression to the levels observed when MAPC were not pre-treated with pro-inflammatory cytokines (Fig. 4b, c). Furthermore, COX-2 inhibition abrogated the enhanced suppression observed when MAPC were pre-treated with TNFα + IL1β (Fig. 4c). Ido-1 inhibitors did not have any reversible effect (Fig. 4b). The inhibitors alone did not have any effect on T-cell proliferation following exposure to MBP (see Additional file 5). Together, these results demonstrate that rMAPC are able to suppress the proliferation of encephalitogenic T cells when restimulated with cognate antigen.

### rMAPC acquire a chemoattractive profile following licensing

**Fig. 5:**
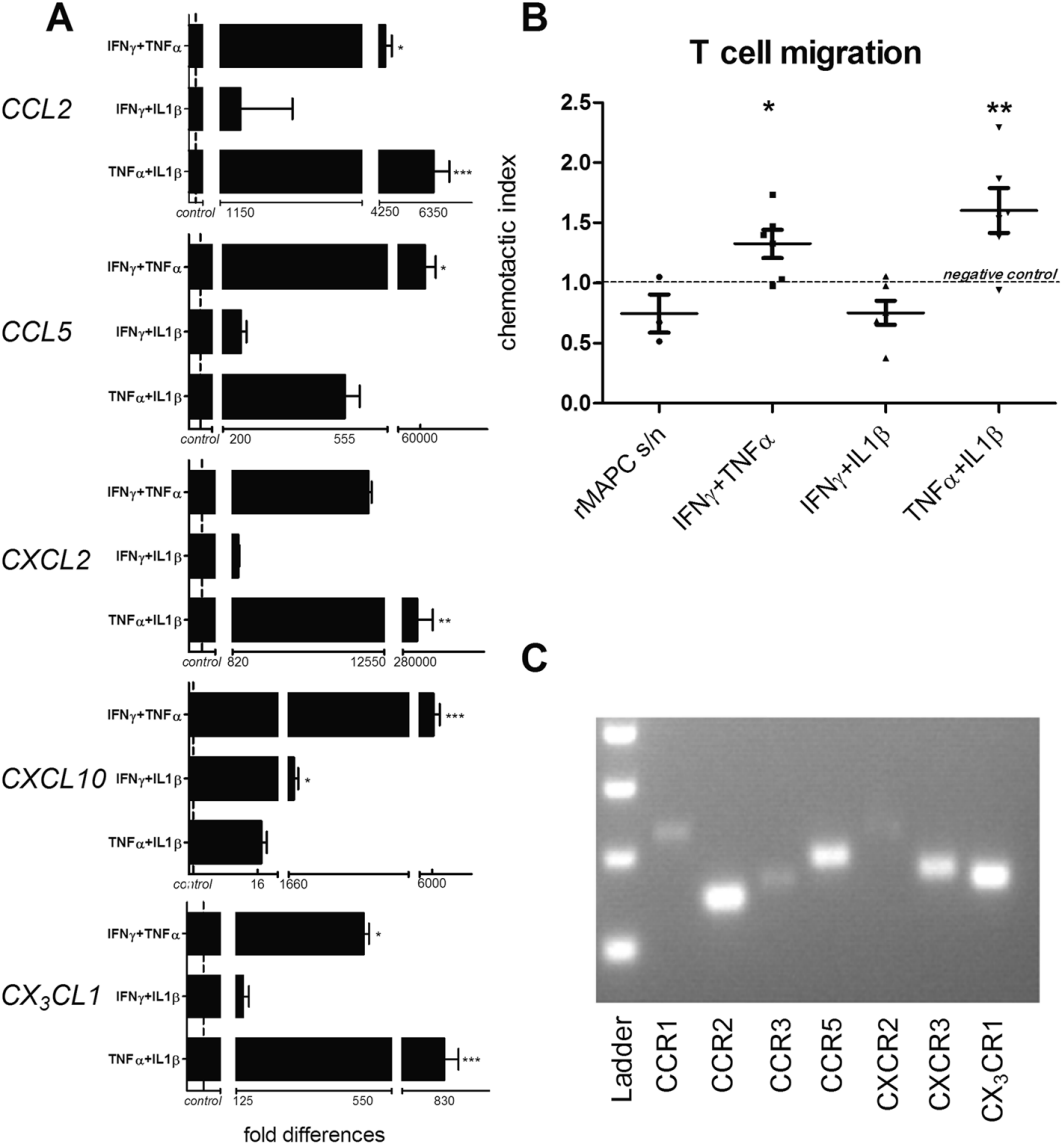
Inflammatory treatment enhances the chemoattractive activity of rMAPC. **a** Gene expression of chemokines by rMAPC following inflammatory treatment. Fold differences compared to control (PBS treated, *dotted line*) are shown. Significant differences with the control condition are indicated with asterisks. Values represent mean ± SEM from five independent experiments. **b** Migration of MBP-specific T cells towards supernatants derived from licensed rMAPC (with defined cytokines combinations). Chemotactic index is shown, which is calculated by dividing the number of migrated T cells per condition by the number of migrated T cells of the negative control (non-conditioned medium). Results are from three independent experiments, with duplicates per experiment. **c** Detection of chemokine receptor mRNA expression in MBP-specific T cells. The genes selected have as ligands the chemokines upregulated in panel **a**. The picture is a representative experiment out of a total of three independent experiments. Non-parametrical Kruskal Wallis multiple groups comparison test was used followed by Dunns test for differences between groups. Significant differences with the control condition are indicated with asterisks: *P ≤ 0.05, **P ≤ 0.01, ***P ≤ 0.001. *IFN* interferon, *IL* interleukin, *rMAPC s/n* supernatant derived by naïve (non-treated) rat multipotent adult progenitor cells, *TNF* tumor necrosis factor

T cell chemotaxis is crucial for the suppression of T-cell proliferation by MSCs [43]. rMAPC constitutively express mRNA copies of certain chemokines that are massively increased after inflammatory treatment. IFNγ + TNFα led to a significant induction of four out of five chemokines tested. TNFα + IL1β upregulated three out of five of the chemokines, while IFNγ + IL1β only significantly induced *CXCL10* (Fig. 5a, Additional file 4).

To confirm the secretion of chemokines in the culture medium following inflammatory treatment, we allowed MBP-specific T cells to migrate towards supernatants of conditioned medium from licensed rMAPC. Enhanced T-cell migration was observed towards supernatant of rMAPC after treatment with TNFα + IL1β and to a lesser extent after IFNγ + TNFα pre-treatment. No significant T-cell migration towards supernatant of IFNγ + IL1β treated rMAPC was observed (Fig. 5b). These results match with the massive upregulation of chemokine mRNA expression (Fig. 5a). Chemokine receptors were all detected on myelin-specific T cells used in the migration assay (Fig. 5c).

We conclude that rMAPC are able to exert chemoattraction towards T cells when challenged with pro-inflammatory stimuli.

### rMAPC express a set of functional chemokine receptors

**Fig. 6:**
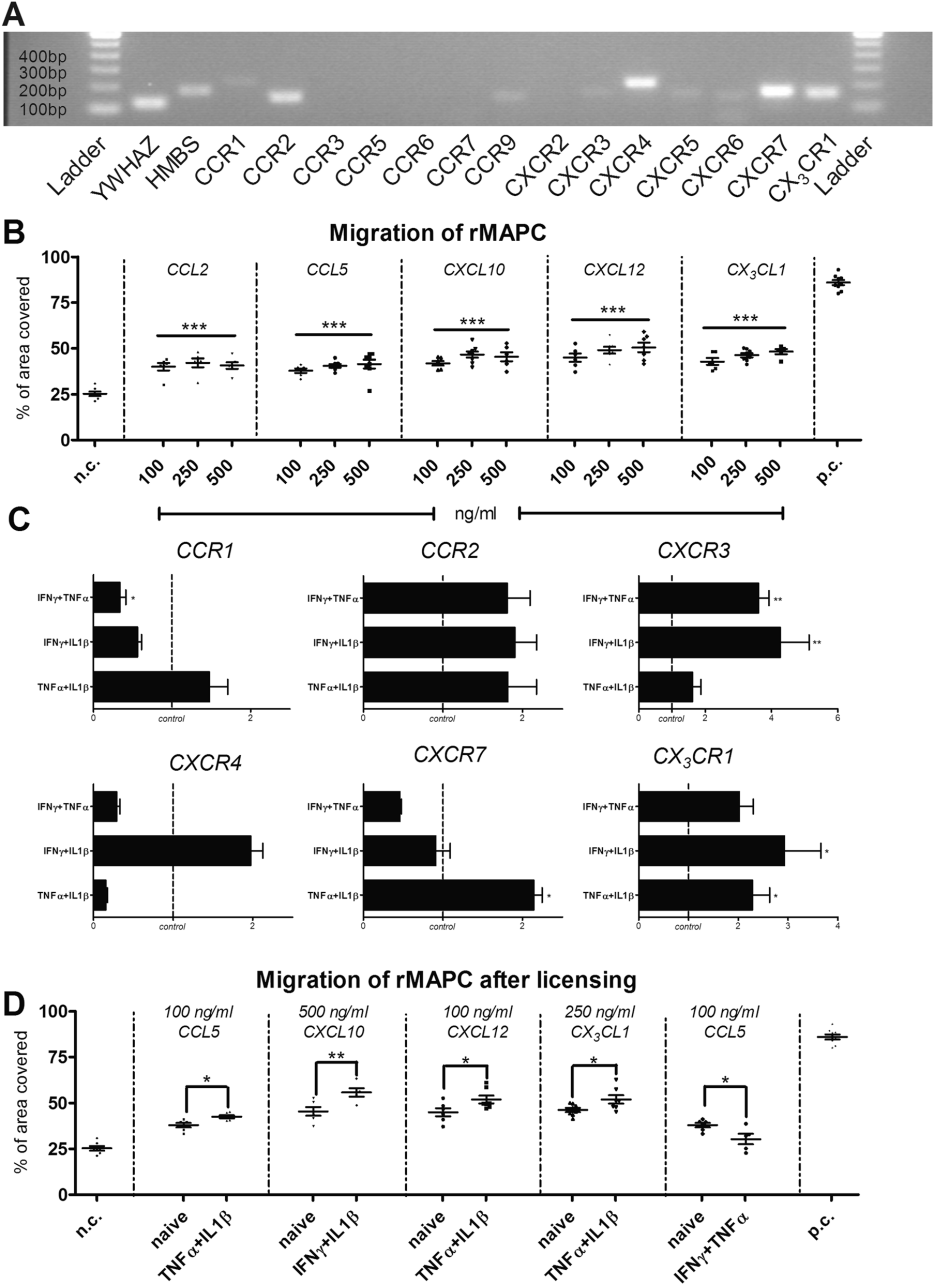
rMAPC migration is altered following licensing. **a** Detection of chemokine receptor mRNA in rMAPC. Amplified cDNAs are visualized in a 1.5 % agarose gel. YWHAZ and HMBS were used as loading controls. One representative experiment out of five is shown. **b** rMAPC migration towards important MS pathogenesis chemokines. Three different concentrations of chemokines were used in the lower compartment (100, 250 and 500 ng/ml). The percentage of area covered by the migrated cells is shown. Values represent mean ± SEM from four independent experiments, with duplicates per experiment. Asterisks show statistical significance between the different concentrations of each chemokine and the negative control. No statistically significant differences were found between the different concentrations for each chemokine. Positive control values showed statistically significant differences with all the other conditions. Data were analyzed with one way analysis of variance comparison test, followed by Dunnett’s test for differences between the groups. **c** Gene expression of chemokine receptors by rMAPC following inflammatory treatment. Fold differences compared to control (PBS treated, *dotted line*) are shown. Significant differences with the control condition are indicated with asterisks. Values represent mean ± SEM from five independent experiments. **d** rMAPC migration following 12 hours of cytokine pre-treatment. Three different concentrations of chemokines were used in the lower compartment (100, 250 and 500 ng/ml). Only the statistically significant changes are shown here. The percentage of area covered by the migrated cells is shown. Values represent mean ± SEM from four independent experiments, with duplicates per experiment. Asterisks show the statistical significant difference between the pre-treated and not treated (naïve) migrated fraction towards specific concentrations of each chemokine. All values differ significantly with n.c. Mean of positive control is statistically significant to all the other conditions. Data were analyzed with one way analysis of variance comparison test, followed by Dunnett’s test for differences between the groups. *P ≤ 0.05, **P ≤ 0.01, ***P ≤ 0.001. *IFN* interferon, *IL* interleukin, *n.c.* negative control, *p.c.* positive control, *rMAPC* rat multipotent adult progenitor cells, *TNF* tumor necrosis factor

Chemokine receptors are potentially involved in MSC homing when injected in pathological conditions [48]. Therefore, we sought to explore the chemokine receptor repertoire of rMAPC. We demonstrate that rMAPC express mRNA levels of *CCR1*, *CCR2*, *CCR9*, *CXCR3*, *CXCR4*, *CXCR5*, *CXCR6*, *CXCR7* and *CX*_*3*_*CR1*. rMAPC did not express detectable levels of *CCR3*, *CCR5*, *CCR6*, *CCR7* and *CXCR2* (Fig. 6a).

To confirm the functionality of the receptors expressed, we explored the migration of rMAPC towards chemokines known to be expressed during neuroinflammation [49]. rMAPC were able to migrate to all chemokines tested, namely CCL2, CCL5, CX3CL1, CXCL12α and CXCL10 (Fig. 6b). Generally we observed a dose-dependent increase in chemotaxis in all chemokines used. These results indicate that rMAPC are attracted to neuroinflammatory chemokines.

### Licensing affects the expression of chemokine receptors genes and the migration of rMAPC

Systemic inflammation in the periphery could potentially affect rMAPC migratory behavior and thus dictate migration towards ongoing inflammation in the CNS and lymphoid organs. Following licensing we saw a differential upregulation of chemokine receptors (Fig. 6c, Additional file 4). Specifically, while IFNγ + TNFα treatment increased only the expression of *CXCR3*, IFNγ + IL1β treatment upregulated *CCR9*, *CXCR3*, *CXCR5* and *CX*_*3*_*CR1*. Finally TNFα + IL1β treatment induced the upregulation of *CXCR7* and *CX*_*3*_*CR1* while it was the only condition where *CCR3* expression was induced (data not shown). Of interest, IFNγ + TNFα significantly downregulated the expression of *CCR1.*

We further assessed the migration of rMAPC following inflammatory treatment. We observed selective alterations in the migration pattern of rMAPC upon licensing. In particular, TNFα + IL1β treatment induced an enhanced migration of rMAPC towards CCL5, CXCL12α and CX_3_CL1, while IFNγ + IL1β enhanced migration towards CXCL10 (Fig. 6d). The observed results correlate partially with the alterations in the expression of *CCR1*, *CXCR7*, *CX*_*3*_*CR1* and *CXCR3* following inflammatory treatment. Of note, IFNγ + TNFα treated rMAPC migrated in a lesser extent towards CCL5, correlating with the significant downregulation in the expression of *CCR1*. The three combinations did not confer significant alterations on rMAPC migration towards the various concentrations of chemokines, besides the ones illustrated in Fig. 6d. These results indicate that the inflammatory milieu may modify the migratory ability of rMAPC thereby affecting the expression of certain chemokine receptors and subsequent migratory activity.

### rMAPC enhance their neuroprotective activity features when challenged with a neurodegenerative environment

**Fig. 7:**
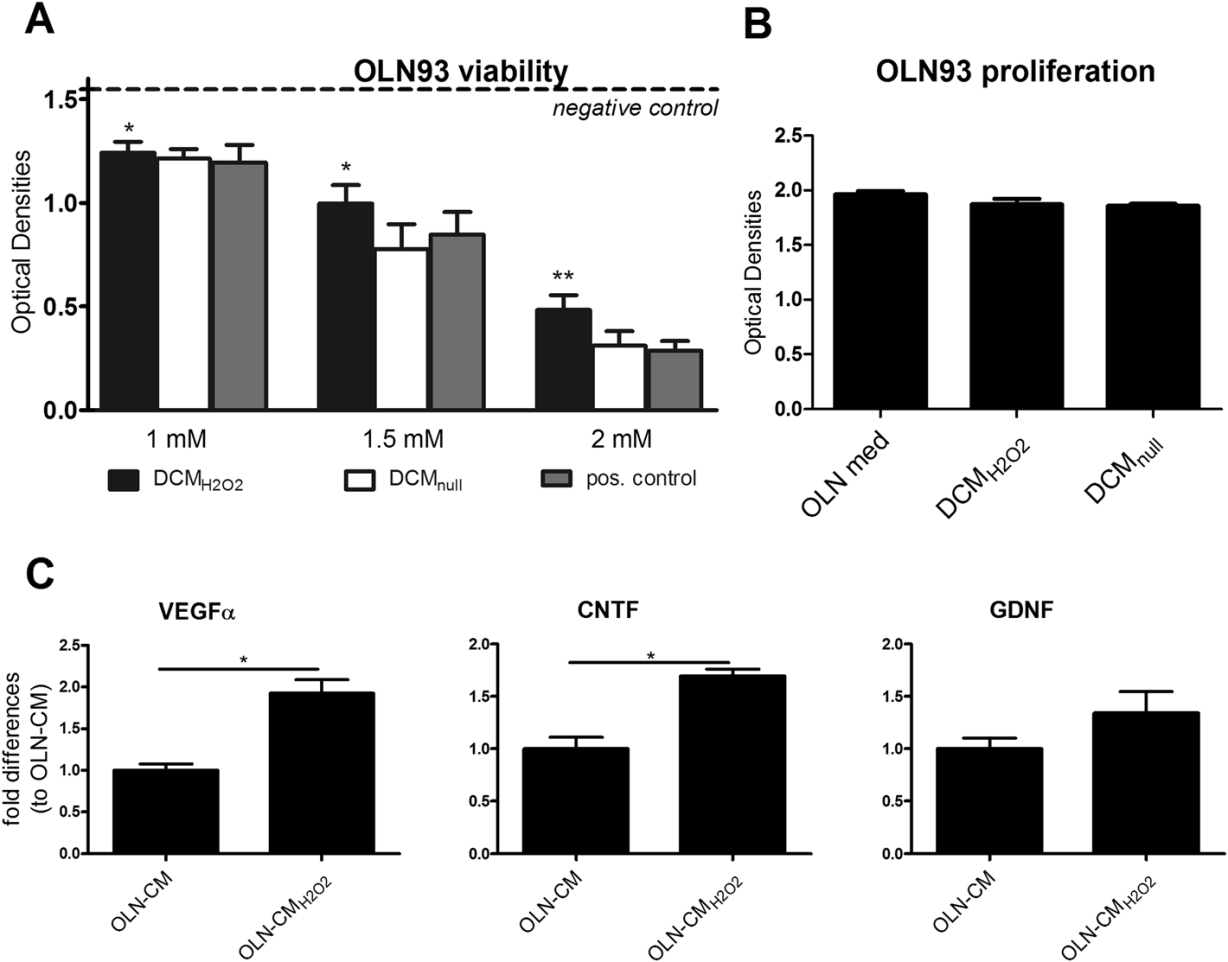
Neurodegeneration enhances the neuroprotective features of rMAPC. **a** Neuroprotection assay where OLN93 cells were pre-treated (with the indicated (double) conditioned media and then three concentrations of H_2_O_2_ were added (pos. control). Optical densities are shown (±SEM) from five experiments. Negative control (*dotted line*) represents OLN93 cells without damage insult. One way analysis of variance followed by Dunnet’s test for multiple comparisons between groups was used. **b** OLN93 cells were pre-treated for 5 hours with rMAPC double conditioned media (DCM) and then allowed to proliferate for 24 hours. Optical densitites are shown (±SEM), n = 5 experiments. **c** rMAPC gene expression of neurotrophic factors (VEGFα, CNTF, GDNF) when treated with OLN-CM_H2O2_ and OLN-CM. Expressions were relatively quantified against the expression of *YWHAZ* and *CycA*. The fold differences are shown in comparison to OLN-CM (±SEM) from five experiments. Mann Whitney *t*-test was used for comparisons between two groups. Significant differences are shown with asterisks: *P ≤ 0.05, ** *P* ≤ 0.01, ***P ≤ 0.001. *CM* conditioned medium, *CNTF* ciliary neurotrophic factor, *GDNF* glial cell line-derived neurotrophic factor, *OLN med* OLN93 medium, *VEGF* vascular endothelial growth factor

Neurodegeneration-induced secretion of growth factors by bone marrow-derived stem cells leads to protection of oligodendrocytes and neurons [34, 44]. To assess potential neuroprotective features of rMAPC in response to neurodegeneration, we challenged rMAPC with secreted factors from the sublethally damaged (H_2_O_2_) oligodendroglia cell line, OLN93 [50]. H_2_O_2_ generates free radicals leading to apoptotic death after oxidative stress, a situation which is prominent within the CNS during neurodegenerative events [51]. We demonstrated that secreted factors released by rMAPC in response to H_2_O_2_-treated OLN93 cells (DCM_H2O2_) are able to partially protect OLN93 cells in all three concentrations of H_2_O_2_ used. rMAPC-derived soluble factors in response to non-damaged OLN93 cells (DCM_null_) showed no evidence of protecting OLN93 cells from H_2_O_2_ damage (Fig. 7a). rMAPC viability under the influence of OLN-CM_H2O2_ was not affected due to possible toxic factors secreted by early apoptotic cells or H_2_O_2_ remnants (data not shown). OLN93 cells did not show any differences in cell proliferation when allowed to proliferate for 24 hours in normal culture medium after the pre-treatment period (Fig. 7b). This finding indicates that the effect of soluble factors secreted by rMAPC is actually protective and does not induce an excessive proliferation resulting in higher cell numbers. Next, we further characterized the behavior of rMAPC in the in vitro generated neurodegenerative environment. We analyzed the mRNA expression of neurotrophic factors known to enhance the survival and proliferation of oligodendrocytes and oligodendrocytes progenitors, as well as those that enhance myelin formation [44, 52–55]. We demonstrate that rMAPC when cultured in supernatant derived from sublethally damaged OLN93 cells (OLN-CM_H2O2_) upregulate the expression of the neurotrophic factors *VEGFα* and *CNTF* but not *GDNF* (Fig. 7c). *HGF* was not detected under any condition. Collectively, these results indicate that the interaction with neurodegenerative signals derived by damaged oligodendrocytes enhances the neuroprotective features of rMAPC.

## Discussion

Stem cell transplantation represents a promising therapeutic approach to treat neuroinflammatory and neurodegenerative disorders such as MS, TBI, SCI and stroke. In this study, we show that rMAPC, a stem cell population similar but distinct to MSCs, possess immunomodulatory and neuroprotective properties which are further enhanced when challenged with selected neuroinflammatory stimuli. These findings point out that MAPC can be considered as a promising therapeutic option for neuroinflammatory and neurodegenerative disorders.

In concordance with other studies, rMAPC exhibit a low immunogenic profile, being negative for co-stimulatory molecules and RT-1b. Expression of RT-1a, though, makes rMAPC prone to lysis by NK cells in case of transplantation as hMAPC [22]. The low immunogenic profile of rMAPC was further confirmed by the low, if any, increase of pro-inflammatory genes following licensing. Upregulation of TNFα by rMAPC should be investigated further, although TNFα can also act as an inhibitory mechanism for CD4 T-cell proliferation [56].

Human and rodent MAPC have been reported to inhibit T-cell proliferation after polyclonal stimulation or allogeneic responses [21, 57, 58]. We demonstrate here that rMAPC effectively suppress proliferation of myelin-reactive T cells *in vitro* and that licensing enhances the suppressive effect of rMAPC. This finding suggests that transplanted MAPC can suppress the autoantigen-specific adaptive inflammatory response in MS. In line with other studies with hMAPC, rMAPC suppressed IFNγ release from autoreactive T cells in co-culture, indicating an attenuation of Th1 polarization [57].

CNS neurodegeneration and peripheral inflammation represent microenvironments that potentially affect the behavior of transplanted cells. We show that pro-inflammatory cytokines markedly increased the expression of immunomodulatory markers previously reported for MSCs and hMAPC, such as iNOS, Ido-1, PD-L1, TSG-6 and prostaglandin E2 (PGE_2_) [23, 39, 40, 42, 43, 59, 60]. IFNγ-driven upregulation of these molecules in MAPC and MSCs collectively has been demonstrated [23, 24, 40, 41, 43, 57]. We now provide evidence that a combination of inflammatory cytokines and, in particular, the combinations of IFNγ/TNFα and TNFα/IL1β induce the increase of iNOS, TSG-6 and COX-2 mRNA transcripts. We demonstrate that the increase of NO and PGE_2_ is functionally involved in the suppression of T-cell proliferation by licensed rMAPC as suppression was abrogated when iNOS and COX-2 were inhibited in the co-cultures.

While PGE2 has already been reported to partially underlie the suppressive effect of mMAPC on T-cell proliferation [21, 41], we now provide evidence that NO is crucial in this process as well. Notably, other studies using mMAPC have excluded PGE_2_ and NO as possible mechanisms for the modulation of alloreactive T-cell responses [21, 61]. Moreover, human and rat MAPC have been reported to modulate alloreactive T-cell responses by an Ido-dependent mechanism [57, 58]. Jacobs et al. showed that IFNγ pre-treatment of hMAPC did not confer an additional suppressive effect as it was already of a great extent without pre-licensing [57]. Overall, there is a pluripotency and species-related variation regarding the mechanisms involved in T-cell suppression, as is apparent for MSCs as well [62, 63].

We found that licensing of rMAPC markedly increased the expression of a large number of chemokines, such as *CXCL2*, *CXCL10*, *CCL2*, *CCL5* and *fractalkine*. These chemokines are well known for their ability to attract encephalitogenic CD4^+^ T cells in the context of neuroinflammation [64–67], as well as CD8+ T cells [68]. The ability of stem cells to migrate towards inflammatory chemokines and to attract leukocytes is a crucial aspect of their potential therapeutic use. For instance, an IFNγ-mediated increase in chemokine release by murine MSCs, such as CXCL10, is key to their immunomodulatory properties [43]. In line with this chemoattractive transcriptional profile, we demonstrated that rMAPC effectively attracted myelin-specific T cells, especially when challenged with IFNγ + TNFα and TNFα + IL1β. In this project we only assessed the capacity of rMAPC to attract T cells. Others have shown that licensing enhances the chemoattractive ability of human MSCs towards other immune cells and, mainly, neutrophils [69]. Future studies should determine whether MAPC also attract other immune cell subsets that play an important role in inflammatory pathophysiology, such as monocytes and dendritic cells. Increased attraction of other immune cell types such as macrophages, for instance, would be crucial for the immunomodulatory mechanisms of MAPC, as it has already been demonstrated that hMAPC suppress their classically activated phenotype in vivo [18].

In addition to their ability to attract T cells, we characterized rMAPC regarding their migratory profile. We observed that rMAPC were able to migrate towards chemokines typical for a neuroinflammatory environment. Of interest, the motility of rMAPC towards these chemokines was further enhanced following licensing, except of the CCL2/CCR1 interaction following IFNγ + TNFα treatment. Overall, these results point out the potential of rMAPC in transplantation experiments where inflammation is already established. Directed injection of NSCs within the CNS in the EAE model showed that inflammation triggered the migration of the transplanted cells towards the white matter tracts, with CXCL12α and CCL2 being important inflamed tissue-derived chemoattractive stimuli [70]. Furthermore, in other neuroinflammatory models, both peripheral- and CNS-targeted injected stem cells are being attracted by local sites of inflammation [71]. While the lack of expression of CCR7 could limit the migration of rMAPC to lymphoid organs, previously reported experimental set up with intravenous administration of hMAPC showed no real obstacle [19]. This points out that other molecules in addition to chemokine receptors are implicated [72]. Overall, these features highlight the migratory potential of rMAPC when injected either in the periphery or within the CNS.

MSCs and NSCs possess neuroprotective features which are induced from inflamed CNS microenvironment [4, 34]. Our findings show that rMAPC that are exposed to sublethally damaged OLN93 cells gain neuroprotective properties. This suggests that damaged oligodendrocytes release mediators that promote the neuroprotective capacity of rMAPC. Similar findings have been reported for rat MSCs [34]. The fact that rMAPC increase their expression of trophic factors confirms this notion. It has already been suggested that human and rodent MAPC provide neuroprotection and vascular regeneration in vivo through the secretion of trophic factors [73–75]. Oligodendrocytes in normal appearing white matter (NAWM) seem to actively participate in immune regulation within the CNS during MS pathology as they are elevating the expression of transcription factors such as STAT-6 and STAT-4, which are important for the activation of anti- and pro-inflammatory pathways, respectively [76]. IL-4 and IL1β, which have been detected in oligodendrocytes in MS NAWM, could prime rMAPC in the same way as do the combinations of pro-inflammatory cytokines. In this way, rMAPC could be effectively triggered by damaged oligodendrocytes or even neurons to secrete trophic factors and thus provide neuroprotection.

## Conclusions

MAPC possess the required properties needed to consider the development of a therapeutic scheme for neuroinflammatory disorders such as MS, TBI and SCI, as was established previously for MSCs [6, 7, 77]. We show that rMAPC are lowly immunogenic and possess numerous potential mechanisms which could facilitate MAPC action, even if they cannot transdifferentiate towards damaged CNS cells. While the microenvironment in neuroinflammatory disorders is likely more complex, we show that typical pro-inflammatory cytokines and mediators released by damaged oligodendrocytes strongly enhance the immunomodulatory properties of rMAPC. Apart from affecting T cells and oligondendrocytes, MAPC may also affect other immune and CNS-resident cell types that are important drivers of neuroinflammation, such as macrophages, microglia and astrocytes. Future studies should define the impact of (licensed) rMAPC on these cell types. Collectively, our findings suggest that MAPC represent an interesting therapeutic tool for the treatment of neuroinflammatory disorders. Yet, future experiments should reinforce the notion that MAPC can reduce neuroinflammation and neurodegeneration in animal models of MS, TBI and SCI [18–20].

## Additional files

Additional file 1:
**Selection of suitable positive control for migration assays.** (TIFF 274 kb) Additional file 2:
**Schematic illustration of the generation of double conditioned media and the neuroprotection assay.** (TIFF 55 kb) Additional file 3:
**Sequences of primers used for quantitative PCR.** (DOCX 18 kb) Additional file 4:
**rMAPC gene expression analysis following inflammatory treatment with real-time PCR.** (DOCX 15 kb) Additional file 5:
**Antigen-specific proliferation of T cells is not affected by the inhibitors alone.** (TIFF 3085 kb)

## Abbreviations

1-Mt-D: 1-methyl-dextro-tryptophan
1-Mt-L: 1-methyl-levo-tryptophan
7AAD: 7 aminoactinomycin D
BSA: Bovine serum albumin
CCL: Chemokine (C-C motif) ligand
CCR: Chemokine (C-C motif) receptor
CD: Cluster of differentiation
cDNA: Complementary deoxyribonucleic acid
CFSE: Carboxyfluorescein succinimidyl ester
CM: Conditioned medium
CNS: Central nervous system
CNTF: Ciliary neurotrophic factor
COX: Cyclooxygenase
CXCL: Chemokine (C-X-C motif) ligand
CXCR: Chemokine (C-X-C motif) receptor
CycA: Cyclophilin A
DCM: Double conditioned medium
DMEM: Dulbecco's modified Eagle's medium
DMSO: Dimethyl sulfoxide
EAE: Experimental autoimmune encephalomyelitis
FACS: Fluorescence-activated cell sorting
FCS: Fetal calf serum
GDNF: Glial cell line-derived neurotrophic factor
H_2_O_2_: Hydrogen peroxide, HGF, Hepatocyte growth factor
hMAPC: Human multipotent adult progenitor cells
HMBS: Hydroxymethylbilane synthase
HO-1: Heme oxygenase 1
Ido-1: iIndoleamine 2,3-dioxygenase 1
IFNγ: interferon gamma
IL1β: Interleukin-1 beta
iNOS: Inducible nitric oxide synthase
L-NMMA: L-N^G^-monomethyl arginine citrate
MAPC: Multipotent adult progenitor cells
MBP: Myelin basic protein
mMAPC: Murine multipotent adult progenitor cells
mRNA: Messenger ribonucleic acid
MS: Multiple sclerosis
MSC: Mesenchymal stem cell
MTT: 3-(4,5)-dimethylthiazol-(−z-y1)-3,5-diphenytetrazoliumronide
NAWM: Normal appearing white matter
NK: Natural killer
NO: Nitric oxide
NSC: Neural stem cell
PBS: Phosphate-buffered saline
PD-L1: Programmed death-ligand 1
PFA: Paraformaldehyde
PGE_2_: Prostaglandin E2
rMAPC: Rat multipotent adult progenitor cells
RPMI: Roswell Park Memorial Institute
RT-PCR: Real time polymerase chain reaction
SCI: Spinal cord injury
SEM: Standard error of the mean
TBI: Traumatic brain injury
TNFα: Tumor necrosis factor alpha
TSG-6: TNF-stimulated gene 6 protein
VEGFα: Vascular endothelial growth factor alpha
YWHAZ: 14-3-3 protein zeta/delta
