# Supplementary figures and images for: Neuroinflammatory signals enhance the immunomodulatory and neuroprotective properties of multipotent adult progenitor cells
Source: Stem Cell Res Ther. 2015 Sep 16;6(1):176. doi: 10.1186/s13287-015-0169-z (PMC4573995; doi:10.1186/s13287-015-0169-z)

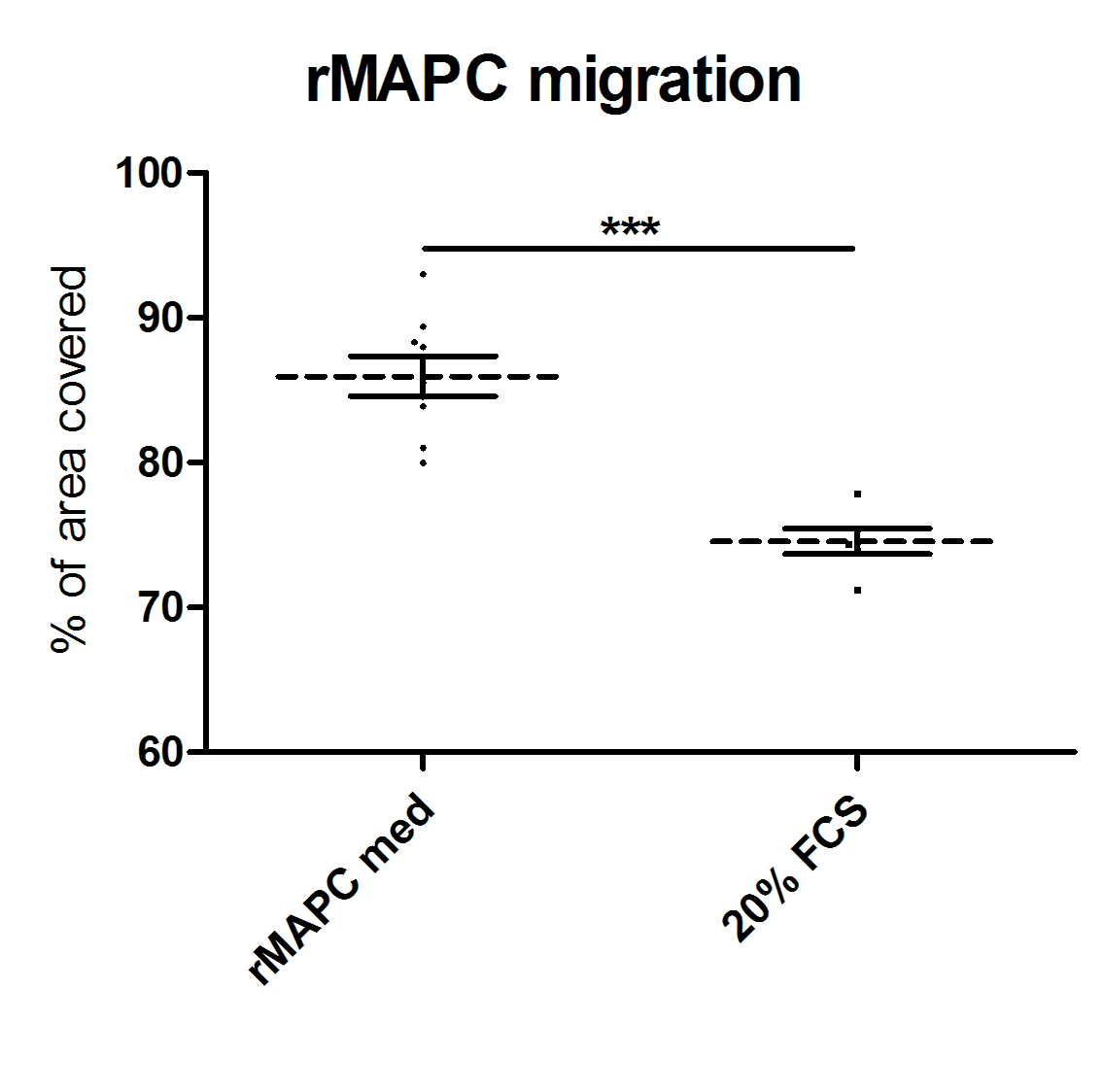

Supplement: Additional file 1: — Selection of suitable positive control for migration assays. (TIFF 274 kb) [file 13287_2015_169_MOESM1_ESM.tif]

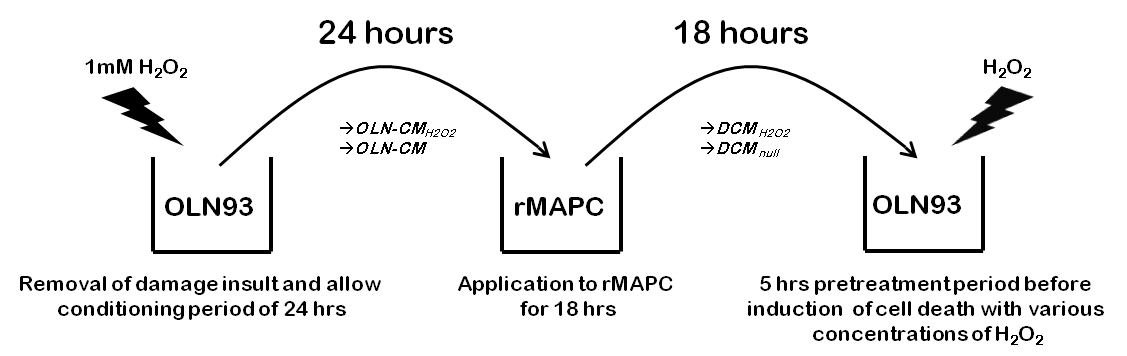

Supplement: Additional file 2: — Schematic illustration of the generation of double conditioned media and the neuroprotection assay. (TIFF 55 kb) [file 13287_2015_169_MOESM2_ESM.tif]

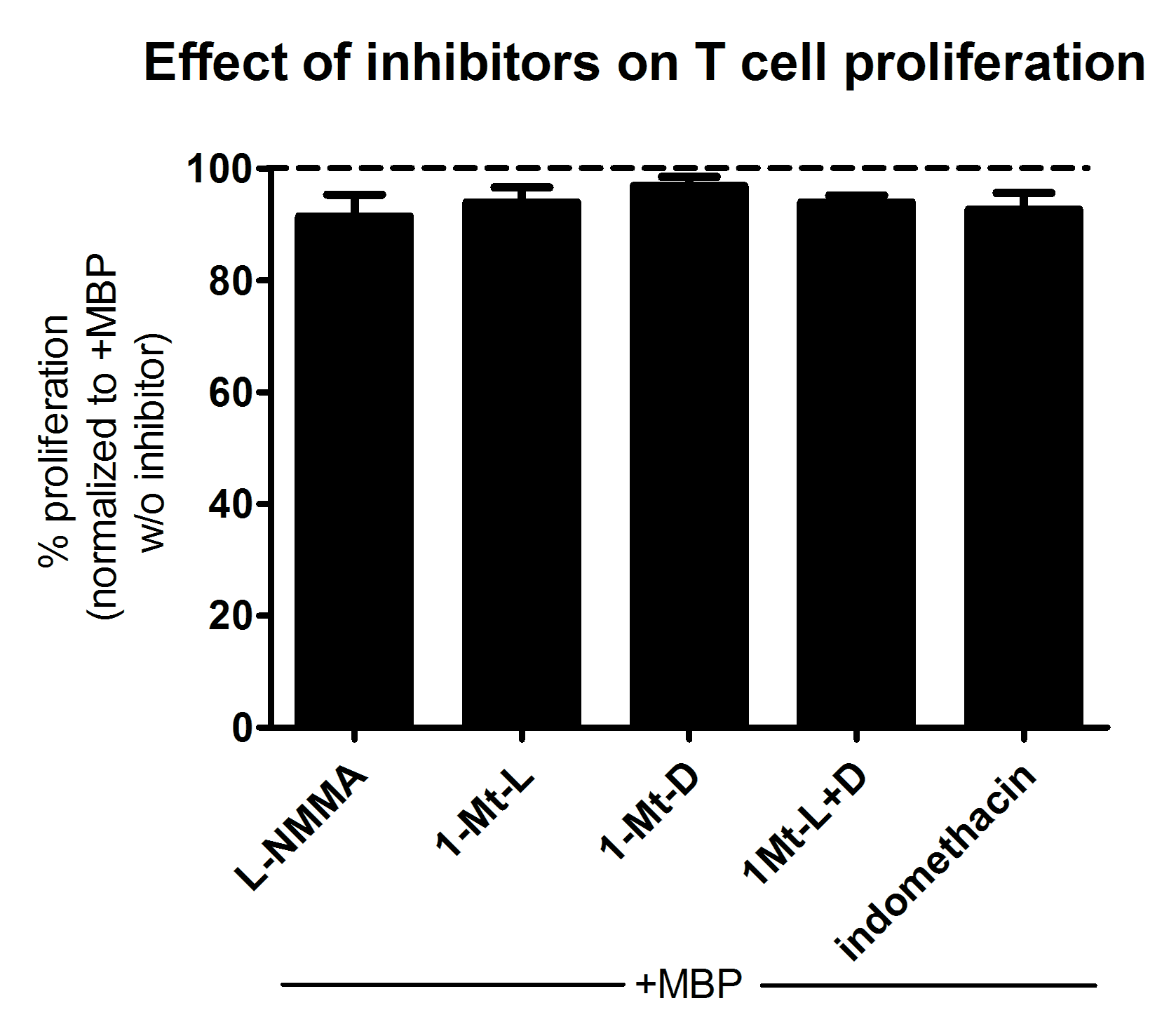

Supplement: Additional file 5: — Antigen-specific proliferation of T cells is not affected by the inhibitors alone. (TIFF 3085 kb) [file 13287_2015_169_MOESM5_ESM.tif]
