# Supplementary material for: Neuroinflammatory signals enhance the immunomodulatory and neuroprotective properties of multipotent adult progenitor cells
Source: Stem Cell Res Ther. 2015 Sep 16;6(1):176. doi: 10.1186/s13287-015-0169-z (PMC4573995; doi:10.1186/s13287-015-0169-z)
Supplement: Additional file 3: — Sequences of primers used for quantitative PCR. (DOCX 18 kb) [file 13287_2015_169_MOESM3_ESM.docx]

Additional file 3: Sequences of primers used for qPCR

| gene | forward primer (5’-3’) | reverse primer (5’-3’) | product (in bp) |
| --- | --- | --- | --- |
| *iNOS* | GCATCCCAAGTACGAGTGGT | TGTTGTAGCGCTGTGTGTCA | 176 |
| *COX-1* | ATGGGAACCAAAGGGAAGAA | GCCTAAGGCCTTGGTAAAGC | 189 |
| *COX-2* | TCACATTTGATTGACAGCCC | CCTTATTTCCTTTCACACCCA | 148 |
| *PD-L1* | ATCATCCCAGAACTGCCTGT | TTCTATCTTCGAAGCCGCAT | 163 |
| *TSG-6* | GCAGCAGGCGTATACCATAGA | TGGAATCCAATCTTTCTGGC | 140 |
| *HO-1* | AGGGAAGGCTTTAAGCTGGT | GAAGGCCATGTCCTGCTCTA | 156 |
| *IDO-1* | CTCCTGCAATCAAAGCAATTC | TGTCTGGATCCACGAAGTCA | 150 |
| *CXCL2* | ATCAGGGTACAGGGGTTGTT | GTTAGCCTTGCCTTTGTTCA | 233 |
| *CXCL10* | GGGCCATAGGAAAACTTGAAATC | CATTGTGGCAATGATCTCAACAT | 71 |
| *CCL2* | TGTCTCAGCCAGATGCAGTT | TTCCTTATTGGGGTCAGCAC | 179 |
| *CCL5* | CCTTGCAGTCGTCTTTGTCA | ATCCCCAGCTGGTTAGGACT | 175 |
| *CX3CL1* | CCATCATCCTGGAGACGAGA | TTGTCCACACGCTTCTCAAA | 142 |
| *CCR1* | TCTGGAAACACAGACTCATTGTC | CCACTCCAATGATAAACACGAA | 227 |
| *CCR2* | TAGGGCTGTGAGGCTCATCT | GGCCTGGTCTAAGTGCATGT | 142 |
| *CCR3* | AAACCTGAGAAGCTAGCCTGTTT | TGCCATTTTACTTGTCTCTGATG | 163 |
| *CCR5* | AAGAAACTCTGGCTCTTGCAG | GGAAGACCATCATGTTACCCA | 185 |
| *CCR6* | CCGGAACATTATTGGAAACG | TAAGGAGTAGGCGATTGGCA | 158 |
| *CCR7* | CCTGGTCATTTTCCAGGTGT | GCCTTAAAGTTCCGCACATC | 129 |
| *CCR9* | TCACAAGCCTTATTCCTGGC | TACAGAGGCGGAAGGAAATG | 148 |
| *CCR10* | ACAGAACAGGTCTCCTGGGG | AGATGGGTGGCCAAGACTAA | 185 |
| *CXCR2* | CATCGTAGAGCTACAGCAGGAT | TCAGCAAAGTCACCAGAACG | 219 |
| *CXCR3* | TGTACCTTGAGGTCAGTGAACG | GGCAGGAAGGTTCTGTCAAA | 166 |
| *CXCR4* | GCCATGGAAATATACACTTCGG | TGTCTGTCATGCTCCTCAGC | 217 |
| *CXCR5* | CGATCTGTACAAGGAACTGGC | TTTCCCATCATACCCAGGAG | 162 |
| *CXCR6* | TGCCCTGCATGTATCTGGTA | AGACAAACACCAGGTCAGCC | 144 |
| *CXCR7* | CGAGGTCACTTGGTTGCTCT | CAGCAGCACATTCTTGTTGG | 162 |
| *CX3CR1* | CAGGACCTCACCATGCCTAC | ACCAGACCGAACGTGAAGAC | 155 |
| *CNTF* | GCAAACACCTCTGACCCTTC | GGCCCTGATGTTTTACATAAGATT | 116 |
| *HGF* | GGTGTTTCACAAGCAATCCA | CTTGTCGGGATATCTTTCCG | 197 |
| *GDNF* | ATTCAAGCCACCATCAAAAG | TCAGTTCCTCCTTGGTTTCG | 220 |
| *VEGFα* | CTGGCTTTACTGCTGTACCTCCACC | GGCACACAGGACGGCTTGAA | 200 |
| *IFNγ* | GAAAGACAACCAGGCCATCAG | TCATGAATGCATCCTTTTTTGC | 101 |
| *TNFα* | CTTATCTACTCCCAGGTTCTCTTCAA | GAGACTCCTCCCAGGTACATGG | 200 |
| *IL6* | TAGTCCTTCCTACCCCAACTTCC | TTGGTCCTTAGCCACTCCTTC | 76 |
| Reference genes |  |  |  |
| *RPL13* | GGATCCCTCCACCCTATGACA | CTGGTACTTCCACCCGACCTC | 131 |
| *TBP* | TGGGATTGTACCACAGCTCCA | CTCATGATGACTGCAGCAAACC | 132 |
| *CycA* | TATCTGCACTGCCAAGACTGAGTG | CTTCTTGCTGGTCTTGCCATTCC | 127 |
| *YWHAZ* | GATGAAGCCATTGCTGAACTTG | GTCTCCTTGGGTATCCGATGTC | 117 |
| *PGK-1* | ATGCAAAGACTGGCCAAGCTAC | AGCCACAGCCTCAGCATATTTC | 104 |
| *HPRT* | CTCATGGACTGATTATGGACAGGAC | GCAGGTCAGCAAAGAACTTATAGCC | 123 |
| *b-actin* | TGTCACCAACTGGGACGATA | GGGGTGTTGAAGGTCTCAAA | 165 |
| *HMBS* | TCCTGGCTTTACCATTGGAG | TGAATTCCAGGTGAGGGAAC | 176 |
