# Supplementary material for: Neuroinflammatory signals enhance the immunomodulatory and neuroprotective properties of multipotent adult progenitor cells
Source: Stem Cell Res Ther. 2015 Sep 16;6(1):176. doi: 10.1186/s13287-015-0169-z (PMC4573995; doi:10.1186/s13287-015-0169-z)
Supplement: Additional file 4: — rMAPC gene expression analysis following inflammatory treatment with real-time PCR. (DOCX 15 kb) [file 13287_2015_169_MOESM4_ESM.docx]

**Additional file 4: rMAPC gene expression analysis with real-time PCR following inflammatory treatment**.

| gene | fold differences^a^ | | | *p* value^b^ |
| --- | --- | --- | --- | --- |
|  | **IFNγ+TNFα** | **IFNγ+IL1β** | **TNFα+IL1β** |  |
| *Molecules involved in immune modulation* | | | | |
| *iNOS* | 116602****** | 32307 | 105176***** | 0.0008 |
| *COX-1* | 0.4038 | 0.9078 | 0.235****** | 0.0010 |
| *COX-2* | 1.457 | 3.553 | 362.4******* | 0.0007 |
| *PD-L1* | 60.02****** | 65.62******* | 0.7924 | 0.0021 |
| *TSG-6* | 20.38 | 2.555 | 34.61******* | 0.0006 |
| *HO-1* | 0.5235 | 0.2588 | 1.039 | 0.0114 |
| *TGFβ1* | 1.216 | 0.8975 | 1.469***** | 0.0045 |
| *Chemokines* | | | | |
| *CXCL2* | 12558***** | 820.4 | 281572****** | <0.0001 |
| *CXCL10* | 6060******* | 1667***** | 16.85 | 0.0005 |
| *CCL2* | 4261***** | 1150 | 6345******* | 0.0005 |
| *CCL5* | 61311******* | 200.8 | 555***** | 0.0005 |
| *CX_3_CL1* | 550.7***** | 123.9 | 827.9******* | 0.0005 |
| *Chemokine receptors* | | | | |
| *CCR1* | 0.3372***** | 0.5585 | 1.478 | 0.0016 |
| *CCR2* | 1.807 | 1.903 | 1.814 | 0.0541 |
| *CCR9* | 1.738 | 3.082****** | 2.358 | 0.009 |
| *CXCR3* | 3.61****** | 4.266****** | 1.618 | 0.0014 |
| *CXCR4* | 0.2891 | 1.974 | 0.1543 | 0.0006 |
| *CXCR5* | 1.964 | 2.737***** | 1.842 | 0.0488 |
| *CXCR6* | 1.209 | 1.45 | 1.127 | 0.2683 |
| *CXCR7* | 0.4596 | 0.9101 | 2.141***** | 0.0011 |
| *CX3CR1* | 2.019 | 2.929***** | 2.289***** | 0.0084 |
| *Pro-inflammatory cytokines* | | | | |
| *IFNγ* | 1.176 | 2.448****** | 1.635 | 0.0097 |
| *TNFα* | 2.202 | 2.914 | 3.325******* | 0.0062 |
| *IL6* | 1.043 | 0.9104 | 1.387 | 0.0630 |

^a^Fold differences compared to the control condition (PBS treated). Expression levels were normalized to the expression of the housekeeping genes *YWHAZ* and *HMBS*. Results are obtained from a total of 5 independent experiments. Differences between groups were tested using Dunns comparison test. Statistical differences compared to control, are indicated with asterisks (* p ≤0.05, **p ≤0.01 and ***p≤0.001 ).

^b^ The *p* values of Kruskal Wallis non parametrical test for comparisons of more than two groups is shown.
